# Predictability of combining Technetium-99m-galactosyl human serum albumin single-photon emission computed tomography/computed tomography and indocyanine green clearance test for posthepatectomy liver failure

**DOI:** 10.1007/s11604-024-01613-4

**Published:** 2024-06-24

**Authors:** Yukinori Okajima, Shin Yanagisawa, Akira Yamada, Tsuyoshi Notake, Akira Shimizu, Yuji Soejima, Yasunari Fujinaga

**Affiliations:** 1 Department of Radiology, Shinshu University School of Medicine, 3-1-1 Asahi, Matsumoto, Nagano, 390-8621 Japan; 2 Medical Data Science Course, Shinshu University School of Medicine, 3-1-1 Asahi, Matsumoto, Nagano, 390-8621 Japan; 3 Division of Gastroenterological, Hepato-Biliary-Pancreatic, Transplantation and Pediatric Surgery, Department of Surgery, Shinshu University School of Medicine, 3-1-1 Asahi, Matsumoto, Nagano, 390-8621 Japan

**Keywords:** Technetium-99m-galactosyl human serum albumin, Single photon emission computed tomography/computed tomography, Plasma clearance rate of indocyanine green, Posthepatectomy liver failure, Liver volume

## Abstract

**Purpose:**

To evaluate the predictive ability of combining Technetium-99m-galactosyl human serum albumin (^99m^Tc‑GSA) single-photon emission computed tomography (SPECT)/computed tomography (CT) volume and plasma clearance rate of indocyanine green (ICGK) for posthepatectomy liver failure (PHLF).

**Materials and methods:**

Fifty patients who underwent ^99m^Tc-GSA scintigraphy as a preoperative examination for segmentectomy or more from July 2021 to June 2023 were evaluated prospectively. Patients were divided into two groups according to the presence or absence of posthepatectomy liver failure (PHLF). Total functional liver volume (t-FLV) and remnant FLV (r-FLV) were measured from ^99m^Tc-GSA SPECT/CT image. Future liver remnant ICGK (ICGK-F) was calculated by ICGK and remnant liver volume from CT. Area under the curve (AUC) of ICGK-F, r-FLV, r-FLV/t-FLV, ICGK × r-FLV, ICGK × r-FLV/t-FLV was calculated to evaluate predictive ability of each parameter for PHLF.

**Results:**

PHLF was occurred in 7 patients. AUC of ICGK × r-FLV was significantly higher than that of ICGK-F (0.99; 95% confidence interval [CI]: 0.96–1 vs 0.82; 95%CI: 0.64–0.96; *p* = 0.036). There was no significant difference between the AUC of r-FLV, r-FLV/t-FLV, ICGK × r-FLV/t-FLV and that of ICGK-F, respectively.

**Conclusion:**

The combination of ^99m^Tc‑GSA SPECT/CT volume and ICGK can predict PHLF more accurately than ICGK-F.

## Introduction

Posthepatectomy liver failure (PHLF) is a serious complication. PHLF is associated with postoperative mortality [1]. PHLF is defined as a condition of impaired residual hepatic excretion and synthetic function due to an inadequate residual hepatic reserve after hepatectomy. Overestimation of residual liver function leads to PHLF, whereas underestimation leads to missed opportunities for patients to undergo hepatectomy, both to the detriment of patients. Therefore, accurate preoperative evaluation of future remnant liver function is important.

Indocyanine green (ICG) clearance test is one of the most reliable quantitative liver function tests. Several studies have reported that the future liver remnant plasma clearance rate of ICG (ICGK-F) is useful for predicting PHLF and mortality after major hepatectomy [2–4]. ICGK-F was calculated from the plasma clearance rate of ICG (ICGK) and the proportion of the remnant liver from CT volumetry. ICGK-F has been used for preoperative evaluation of hepatectomy; however, neither ICGK nor CT volumetry can be used to evaluate the heterogeneity of liver function. Therefore, this index may not precisely evaluate remnant liver function, especially when heterogeneity of liver function exists. Furthermore, the ICG clearance test mainly reflects the excretory function rather than the synthetic function of the liver. Therefore, the prediction of PHLF can be improved by combining the ICG clearance test with an additional quantitative function test that can reflect the synthetic function of the liver.

Technetium-99m-galactosyl human serum albumin (^99m^Tc-GSA) is an asialoglycoprotein analog that specifically binds to the asialoglycoprotein receptor (ASGPR). ASGPR are expressed on the surface of hepatocytes, and the number of ASGPR decreases in patients with liver damage [5, 6]. The degree of ^99m^Tc-GSA accumulation in the liver correlates with the degree of liver damage [7, 8], functional hepatocellular mass, and total hepatocytes number [9]. Furthermore, a small remnant liver volume has been reported as an independent predictive factor for coagulation derangement [10]. Therefore, ^99m^Tc‑GSA scintigraphy can be used to quantitatively evaluate synthetic liver function, in addition to the ICG clearance test, for the prediction of PHLF.

In ^18^F-fluorodeoxyglucose (^18^F-FDG) positron emission tomography/computed tomography (PET/CT), metabolic tumor volume (MTV) has been reported to be useful in predicting tumor prognosis [11–16]. MTV was measured by contouring the tumor margin, which was determined by standardized uptake value (SUV) threshold value. In addition, recent studies have reported the utility of volume based on the SUV threshold, which is similar to MTV, in single-photon emission computed tomography (SPECT)/computed tomography (CT) [17–19]. Volume based on SUV threshold reflects the degree of accumulation and heterogeneity of the target. Therefore, we hypothesize that the combination of ICGK as quantitative excretion liver function index and liver volume based on SUV threshold of ^99m^Tc‑GSA SPECT/CT reflecting heterogeneity and synthetic of liver function can more accurately predict PHLF compared with ICGK-F. To the best of our knowledge, no study has evaluated the remnant liver reserve for the prediction of PHLF by combining volume based on the SUV threshold of ^99m^Tc‑GSA SPECT/CT and the ICG clearance test.

This study aimed to evaluate the predictive ability of the combining ^99m^Tc‑GSA SPECT/CT volume and ICGK for PHLF.

## Materials and methods

### Patients

**Fig. 1:**
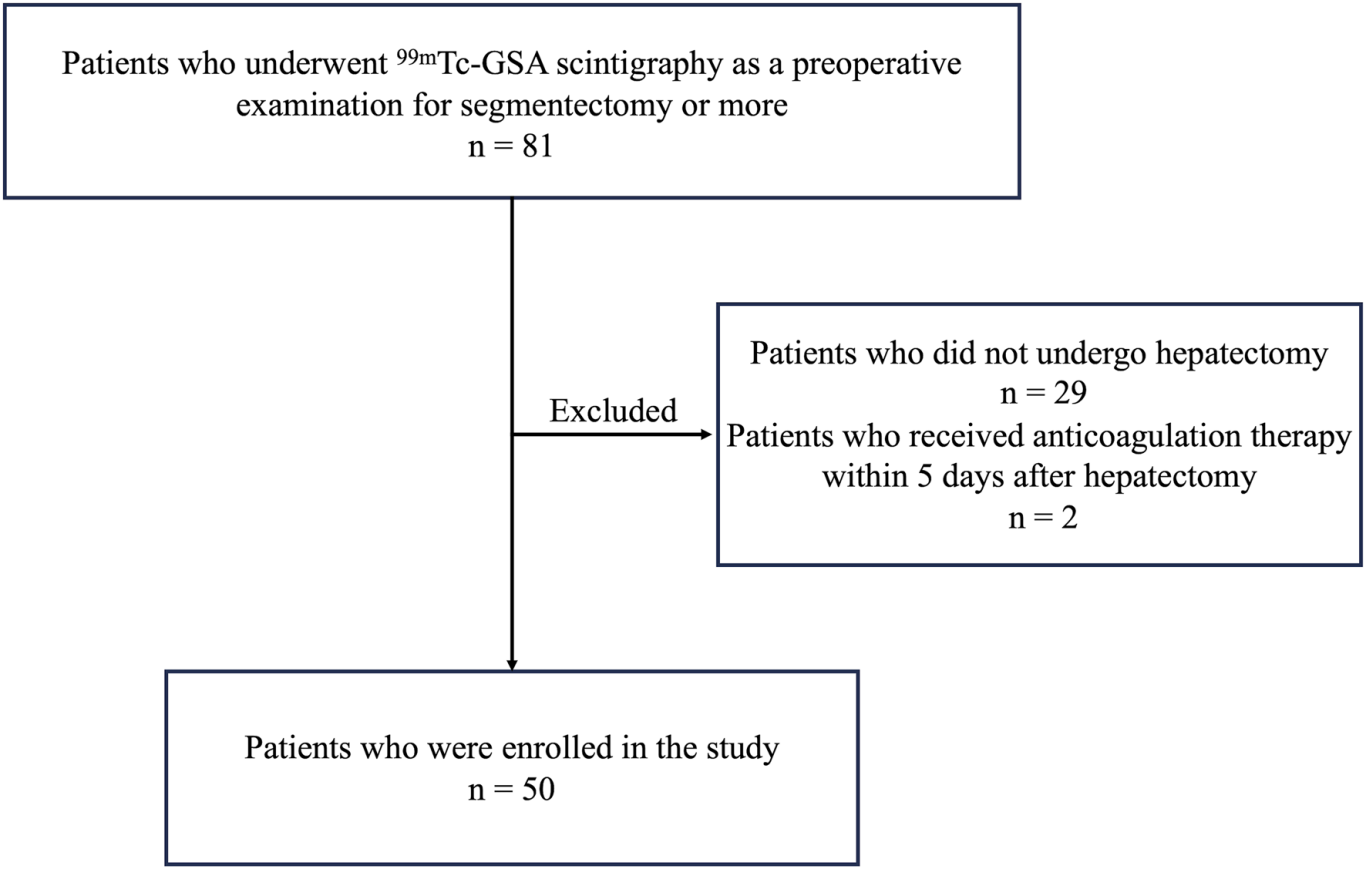
Flowchart of the study. ^*99m*^*Tc-GSA* Technetium-99m-galactosyl human serum albumin

This prospective study was approved by the Institutional Review Board of our institution (No. 5215). Written informed consent was obtained from all participants. Between July 2021 and June 2023, 81 consecutive patients who underwent ^99m^Tc-GSA scintigraphy as preoperative examination for hepatectomy were enrolled in this study. The initial inclusion criteria were as follows: (1) patients who underwent segmentectomy or more and (2) patients who underwent ICG clearance test as a preoperative examination and contrast-enhanced CT as pre-and postoperative examinations. From the initial population, patients who met the following criteria were excluded from the analysis: (1) patients who did not undergo hepatectomy after preoperative examination and (2) patients who had reasons that might affect the PHLF criteria, as described below (Fig. 1).

### Imaging protocols of ^99m^Tc-GSA SPECT/CT

SPECT/CT images were obtained using an SPECT/CT scanner (Symbia T6, Siemens Healthineers, Erlangen, Germany) with a dual-head gamma camera and low-energy, high-resolution collimators. After an overnight fast, ^99m^Tc-GSA (Nihon Medi-Physics Co., Ltd., Tokyo, Japan) at a mean dose of 266.1 MBq (range 123.4–304.9 MBq) was injected intravenously. SPECT images (step-and-shoot, 90 steps of 25 s/step, 360°, and 128 × 128 matrix) were obtained 20 min after ^99m^Tc-GSA injection. A low-dose CT scan (helical, 130 keV, 70 mAs, and 2.5 mm slice thickness) was performed for attenuation correction. SPECT and CT images of the entire liver were obtained. The SPECT images were reconstructed using an ordered subset expectation maximization algorithm (five subsets, nine iterations) and a Gaussian filter (FWHM = 8.0 mm).

### Image analysis of ^99m^Tc-GSA SPECT/CT

**Fig. 2:**
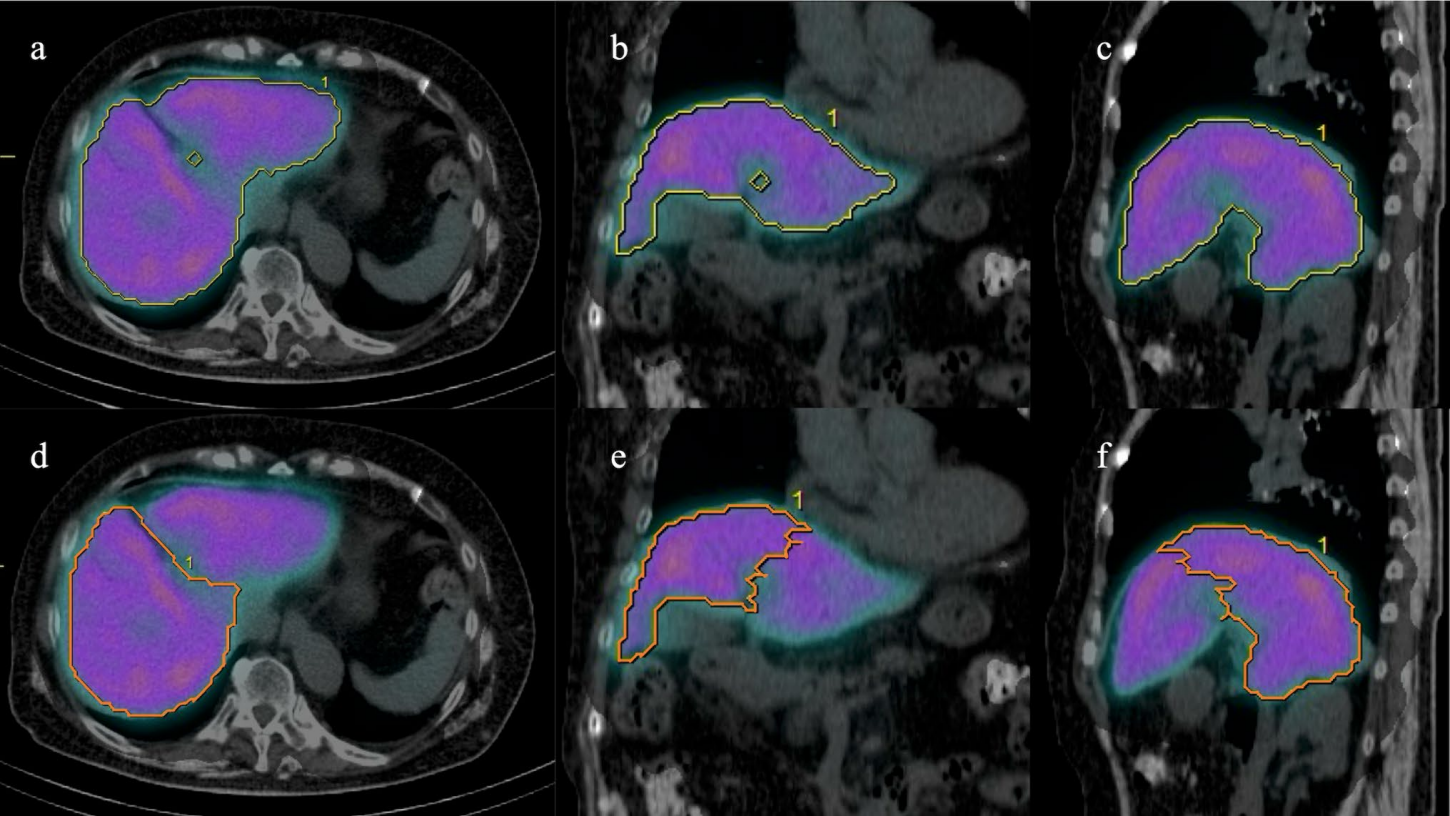
Calculation of functional liver volume of total liver (t-FLV) and functional liver volume of remnant liver (r-FLV) for left lateral sectionectomy. **a** (Transaxial image), **b** (coronal image), and **c** (sagittal image) show calculation of t-FLV (yellow line). The software (RAVAT) automatically contoured whole liver above standardized uptake value (SUV) ≥ 20. **d** (Transaxial image), **e** (coronal image), and **f** (sagittal image) show calculation of r-FLV (orange line). The resection line was drawn manually

The SPECT/CT images were analyzed using the free software RAVAT version 1.00 (Nihon Medi-Physics Co., Ltd., Tokyo, Japan). First, a cubic volume of interest (VOI) containing the entire liver was created. Then, the software automatically detected the region of voxel with SUV ≥ 20. The threshold was determined to match liver contours. SUV was calculated using the following formula: SUV = total radioactivity of the VOI (Bq/mL)/[injected dose (Bq)/body weight (g)]. The software automatically measured liver volume segmented above threshold (SUV ≥ 20) (Fig. 2). Liver volume was defined as the functional liver volume of total liver (t-FLV).

**Fig. 3:**
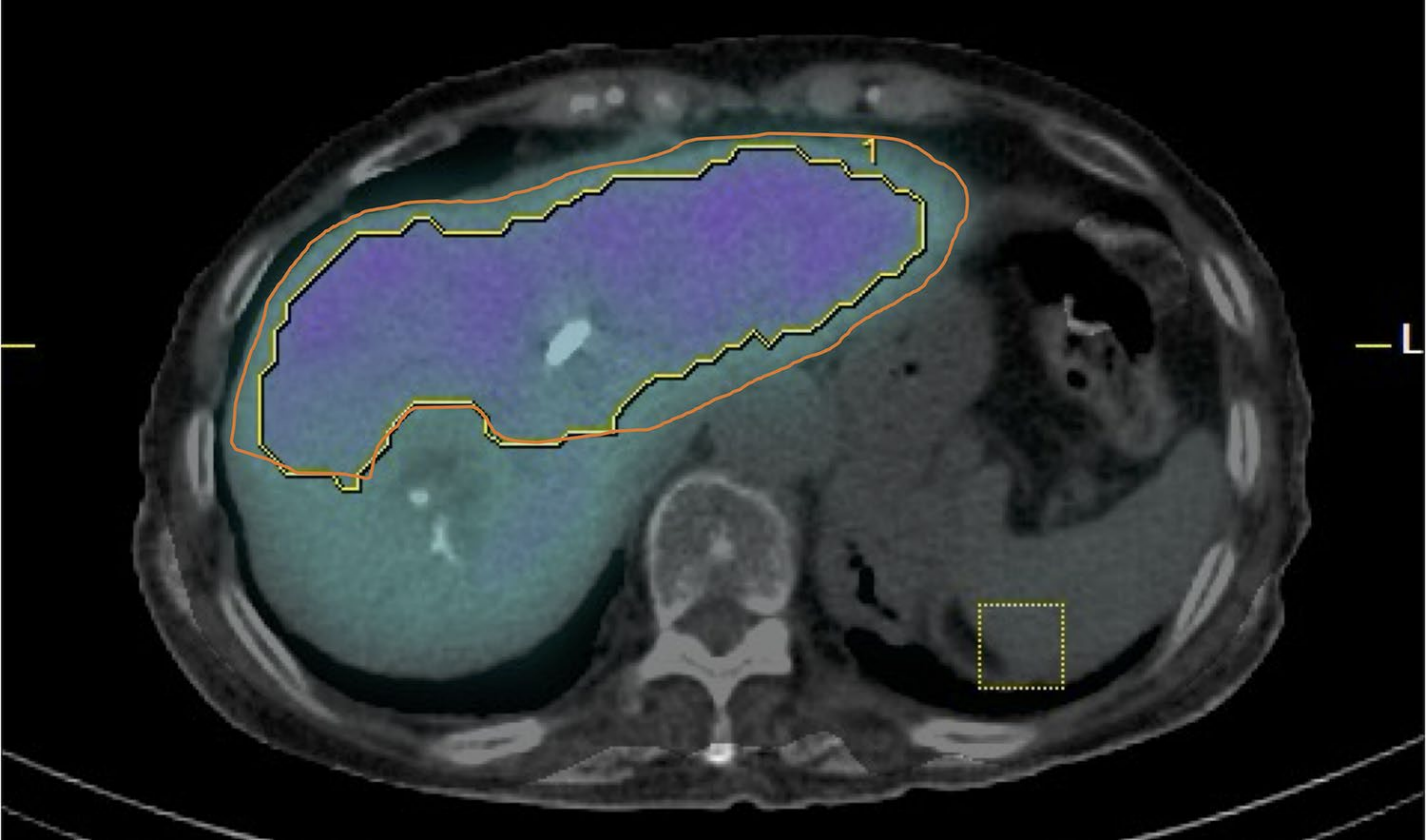
Difference between single-photon emission computed tomography (SPECT) and CT contours in case of right hemihepatectomy after portal vein embolization for right branch of portal vein. In this case, liver accumulation was low, and SPECT contour of remnant liver (yellow line) was located inside CT contour (orange line)

The resection line was determined with reference to the hepatic portal and hepatic veins on SPECT/CT images and pre-and postoperative contrast-enhanced CT images. The resection line was determined by consensus of two board-certified radiologists included in the study (Y.O., 11 years of radiology experience; S.Y., 22 years of radiology experience). Based on the resection line, the FLV of the remnant liver (r-FLV) was measured using RAVAT (Figs. 2 and 3).

Radioactivity in the syringe before and after injection, the time before and after injection, and body weight were recorded to calculate the SUV. A cross-calibration factor was calculated using a phantom to convert the SPECT count into a radioactivity value.

### Imaging protocols and analysis of CT

Dynamic contrast-enhanced CT was performed using a 256-row CT scanner (Revolution CT, GE Healthcare, Chicago, IL, USA). The tube voltage was 120 kVp and the reconstruction thickness was 1.25 mm. A nonionic iodinated contrast agent (Iopamiron 370, Bayer Healthcare, Berlin, Germany) was administered intravenously. The total dose of the contrast agent was 100 ml and the injection rate was 3 ml/s. Images were obtained before injection and 34, 40, 46, 67, and 135 s after injection initiation. One patient received pre-injection and 40 and 135 s after injection initiation. Images at 67 s or 135 s after injection were used to determine resection line, because portal vein and hepatic vein were clearly enhanced. Total liver volume (t-LV) and remnant liver volume (r-LV) were measured using the AW Server software, version 3.2 (GE Healthcare, Chicago, IL, USA).

### ICG clearance test

The ICG clearance test was performed as follows. A dose of 0.5 mg/kg ICG was administered intravenously, and blood was sampled before and at 5, 10, and 15 min after ICG administration. ICGK was determined using regression analysis.

ICGK-F was calculated using the following formula: ICGK × r-LV/t-LV. In addition, the ICGK × r-FLV and ICGK × r-FLV/t-FLV ratios were calculated.

### Preoperative and postoperative data

Etiology of liver disease was obtained from electronic medical record. Preoperative serum levels of total bilirubin, albumin, aspartate aminotransferase, alanine aminotransferase, international normalized ratio of prothrombin time (PT-INR), and platelet counts were recorded. Postoperative serum total bilirubin and PT-INR levels were recorded daily after hepatectomy. The type of hepatectomy, the results of the pathological diagnosis, and presence or absence of cirrhosis in the background liver were recorded.

### Preoperative management

If the future remnant liver volume was insufficient, portal vein embolization (PVE) or associated liver partition and portal vein ligation for staged hepatectomy (ALPPS) was performed. PVE was performed when future remnant liver volume was less than 40% of the total liver volume on CT. ALPPS was performed when the future remnant liver volume was less than 30% of the total liver volume on CT volumetry, and the interval to hepatectomy was shortened. ^99m^Tc-GSA scintigraphy, ICG clearance test, and contrast-enhanced CT were repeated within 1 month after PVE or ALPPS. Hepatectomy was planned again if the future remnant liver volume was > 40% of the total liver volume on the CT. Contrast-enhanced CT and SPECT/CT of post-PVE or ALPPS were used to calculate t-LV, r-LV, t-FLV, and r-FLV.

### Definition of PHLF

PHLF was defined according to the criteria of the International Study Group of Liver Surgery (ISGLS) as increased PT-INR and hyperbilirubinemia on or after postoperative day 5 [20]. The cut-off values of PT-INR and serum total bilirubin level at our institute were 1.15 and 1.50 mg/dl, respectively. If the PT-INR or serum total bilirubin level increased preoperatively, PHLF was defined as increased PT-INR and hyperbilirubinemia on or after postoperative day 5 (compared with the values on the previous day). The severity of PHLF was graded based on its impact on clinical management. Grade A was defined as PHLF that did not require any changes in the clinical management of the patient. Grade B was defined as PHLF requiring noninvasive treatment. Grade C was defined as PHLF requiring an invasive treatment.

### Statistical analysis

Continuous variables were expressed as medians (interquartile ranges). Continuous variables were compared between the PHLF and non-PHLF groups using the Mann–Whitney U test, and categorical variables were compared using Fisher’s exact and Chi-squared tests. Receiver-operating characteristic (ROC) curves were derived, and the area under the curve (AUC) was calculated to examine the diagnostic performance for predicting PHLF in ICGK-F, r-FLV, r-FLV/t-FLV, ICGK × r-FLV, and ICGK × r-FLV/t-FLV, respectively. The cut-off value was determined as the point closest to the upper-left corner of the ROC curve. The sensitivity and specificity of the PHLF were calculated using this cut-off value. Sensitivity and specificity for increased PT-INR or hyperbilirubinemia after hepatectomy in ICGK-F, r-FLV, and ICGK × r-FLV were calculated using the cut-off value for PHLF to examine whether synthetic reserve or excretory reserve was more influential in each index predicting PHLF. The bootstrap method with 2000 bootstrap samples was used to compare the area under the curve (AUC) values and to calculate the 95% confidence interval (CI) for sensitivity and specificity.

Statistical significance was considered when *p* value was less than 0.05 or 95%CI did not overlap. Statistical analysis was performed using the Bell Curve for Excel (Social Survey Research Information Co., Ltd., Tokyo, Japan) and R version 4.2.2.

## Results

Of the 81 enrolled patients, 31 were excluded from the analysis. Of the 31 excluded patients, 29 were excluded, because they did not undergo hepatectomy for several reasons (e.g., detection of metastases or bilobar liver lesions on preoperative CT or MRI, and suspected benign lesions on preoperative CT or MRI). Two patients were excluded, because they received anticoagulation therapy within 5 days after hepatectomy and PHLF was not adequately evaluated by PT-INR.

Fifty patients underwent segmentectomy or more. The median (range) interval between the preoperative evaluation (^99m^Tc-GSA scintigraphy, ICG clearance test, and contrast-enhanced CT) and hepatectomy was 20.5 (4–124) days. The types of hepatectomy included segmentectomy in 6 patients, sectionectomy in 11, hemihepatectomy in 31, and trisectionectomy in 2. In six patients, partial resection was performed in addition to the surgical procedures described above. PVE and ALPPS were performed in 5 patients (PVE in 3 patients and ALPPS in 2 patients). PHLF occurred in 7 patients (grade A in 5 patients and grade B in 2 patients). The patient characteristics according to the presence or absence of PHLF are shown in Table 1.

**Table 1:** Patient characteristics

	PHLF (*n* = 7)	No PHLF (*n* = 43)	*p* Value
Age (years)	73 (66–81)	68 (52–75)	0.15
Gender (male/female)	5/2	30/13	1.000
Preoperative laboratory data			
Total bilirubin (mg/dL)	1.04 (0.87–1.49)	0.77 (0.66–0.95)	0.063
Albumin (g/dL)	4.0 (3.3–4.3)	3.9 (3.6–4.2)	0.93
AST (units/L)	25 (25–31)	22 (18–35)	0.17
ALT (units/L)	23 (15–34)	20 (13–35)	0.68
Platelet count (× 10^4^/μL)	20.8 (17.6–21.9)	20.1 (18.7–25.0)	0.79
PT-INR	1.08 (1.08–1.12)	1.03 (1.00–1.08)	0.017
Liver cirrhosis (yes/no)	0/7	4/39	1.000
Etiology of liver disease			0.24
Hepatitis B	1	3	
Hepatitis C	0	2	
Alcohol	0	1	
NASH	0	4	
PBC	1	0	
Autoimmune hepatitis	0	1	
Others	5	32	
PVE or ALLPS (yes/no)	2/5	3/40	0.13
Type of hepatectomy			0.49
Segmentectomy	1	5	
Sectionectomy	1	10	
Hemihepatectomy	4	27	
Trisectionectomy	1	1	
Diagnosis			0.86
Hepatocellular carcinoma	1	10	
Cholangiocarcinoma	2	12	
Liver metastasis	2	8	
Liver transplant donor	1	4	
Intraductal papillary neoplasm of bile duct	0	5	
Hemangioma	1	3	
Angiomyolipoma	0	1	

Continuous variables are shown as median (interquartile range)

*AST* aspartate aminotransferase, *ALT* alanine aminotransferase, *PT-INR* international normalized ratio of prothrombin time, *NASH* non-alcoholic steatohepatitis, *PBC* primary biliary cholangitis, *PVE* portal vein embolization, *ALLPS* associating liver partition and portal vein ligation for staged hepatectomy

**Fig. 4:**
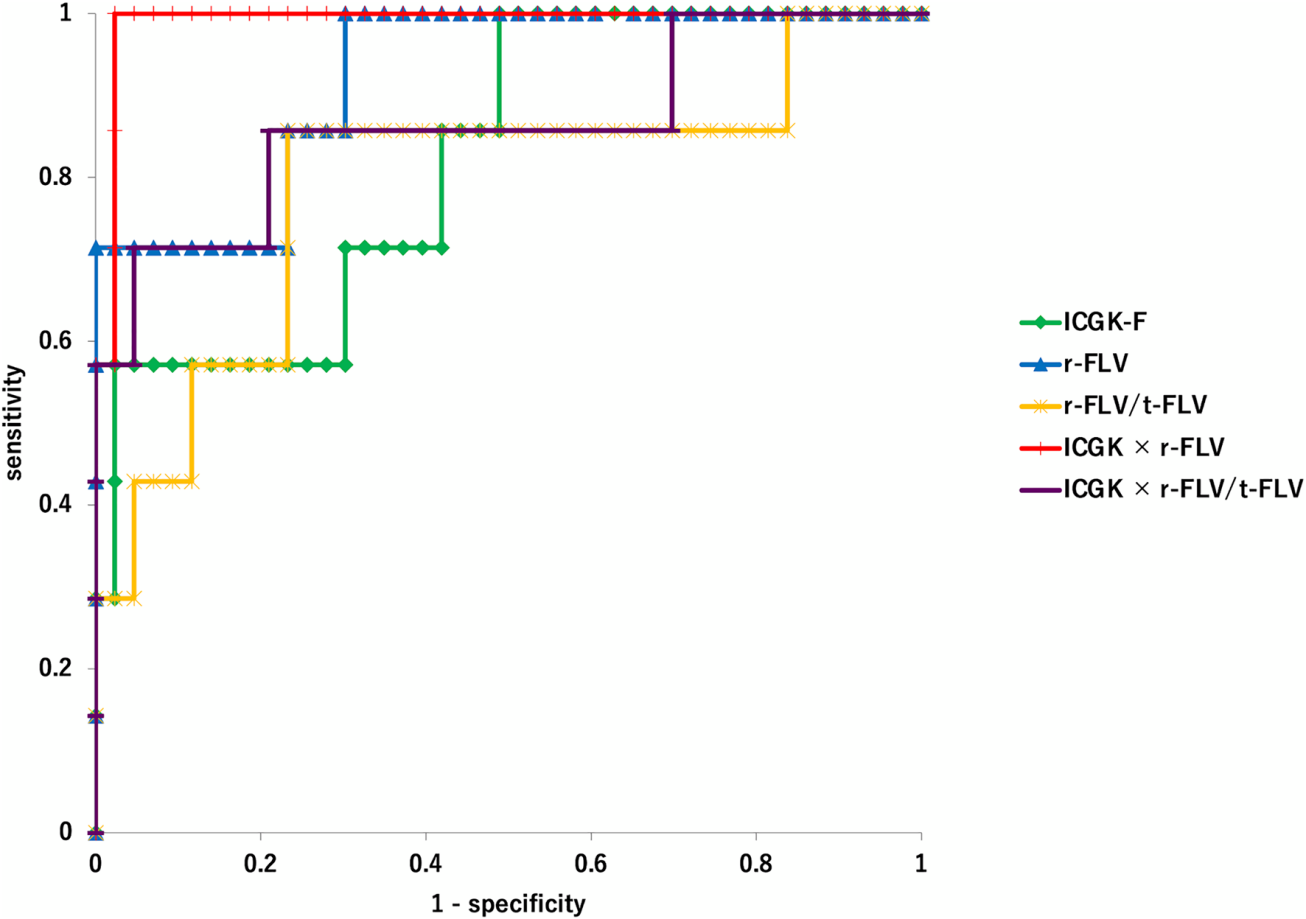
Receiver-operating characteristic curve for predicting PHLF. *PHLF* posthepatectomy liver failure, *ICGK* plasma clearance rate of indocyanine green, *ICGK-F* future liver remnant plasma clearance rate of indocyanine green, *t-FLV* functional liver volume of total liver, *r-FLV* functional liver volume of remnant liver

ICGK-F, r-FLV, r-FLV/t-FLV, ICGK × r-FLV, and ICGK × r-FLV/t-FLV were compared based on the presence or absence of PHLF (Table 2). All the parameters were significantly lower in the PHLF group than in the non-PHLF group. The AUC values, cut-off values, sensitivity, and specificity of these parameters are summarized in Table 3. The AUC of ICGK × r-FLV (0.99, 95%CI: 0.96–1.00) was significantly greater than that of ICGK-F (0.82, 95%CI: 0.64–0.96) without overlap in 95%CI. There were no significant differences among the AUC of r-FLV, r-FLV/t-FLV, ICGK × r-FLV/t-FLV, and ICGK-F. The receiver-operating characteristic (ROC) curves for each parameter are shown in Fig. 4.

**Table 2:** Comparison of ICGK-F, r-FLV, r-FLV/t-FLV, ICGK × r-FLV, and ICGK × r-FLV/t-FLV

	PHLF (*n* = 7)	No PHLF (*n* = 43)	*p* Value
ICGK-F	0.065 (0.060–0.105)	0.122 (0.089–0.150)	0.007
r-FLV	395.614 (364.633–496.54)	732.631 (572.284–834.935)	< 0.001
r-FLV/t-FLV	0.539 (0.378–0.621)	0.737 (0.651–0.811)	0.014
ICGK × r-FLV	63.60 (57.34–72.56)	122.32 (104.44–162.28)	< 0.001
ICGK × r-FLV/t-FLV	0.072 (0.058–0.092)	0.127 (0.101–0.159)	0.002

Continuous variables are shown as median (interquartile range)

*PHLF* posthepatectomy liver failure, *ICGK* plasma clearance rate of indocyanine green, *ICGK-F* future liver remnant plasma clearance rate of indocyanine green, *t-FLV* functional liver volume of total liver, *r-FLV* functional liver volume of remnant liver

**Table 3:** Predictive ability of ICGK-F, r-FLV, r-FLV/t-FLV, ICGK × r-FLV, and ICGK × r-FLV/t-FLV for PHLF

	AUC	Cutoff value	Sensitivity (%)	Specificity (%)	*p* Value
ICGK-F	0.82 (0.64–0.96)	0.096	71.4 (28.6–100)	70.0 (55.8–83.7)	
r-FLV	0.92 (0.81–1)	555.585	85.7 (57.1–100)	76.7 (65.1–88.4)	0.31
r-FLV/t-FLV	0.79 (0.56–0.96)	0.62	85.7 (57.1–100)	76.7 (65.1–88.4)	0.71
ICGK × r-FLV	0.99 (0.96–1)	80.248	100 (100–100)	97.7 (93.0–100)	0.036
ICGK × r-FLV/t-FLV	0.86 (0.65–1)	0.0984	85.7 (57.1–100)	79.1 (67.4–90.7)	0.54

The figures in parentheses refer to 95% confidence interval. *P* values indicate comparison of AUC values with ICGK-F

*AUC* area under the curve, *PHLF* posthepatectomy liver failure, *ICGK* plasma clearance rate of indocyanine green, *ICGK-F* future liver remnant plasma clearance rate of indocyanine green, *t-FLV* functional liver volume of total liver, *r-FLV* functional liver volume of remnant liver

The sensitivity and specificity of ICGK-F and ICGK × r-FLV for increased PT-INR and hyperbilirubinemia are summarized in Table 4. Using the cut-off values for PHLF, increased PT-INR and hyperbilirubinemia were observed in 11 and 12 patients, respectively. Specificity of ICGK × r-FLV for increased PT-INR (97.4%, 95%CI: 92.3–100%) and hyperbilirubinemia (97.4%, 95%CI: 92.1–97.4%) were significantly higher than those of ICGK-F (74.4%, 95%CI: 64.5–87.2% for increased PT-INR; 71.1%, 95%CI: 55.3–84.2% for hyperbilirubinemia) and r-FLV (79.5%, 95%CI: 66.7–92.3% for increased PT-INR; 73.7%, 95%CI: 60.5–86.8% for hyperbilirubinemia). There were no significant differences in the sensitivities of ICGK-F, r-FLV, and ICGK × r-FLV for increased PT-INR and hyperbilirubinemia.

**Table 4:** Predictive ability of ICGK-F and ICGK × r-FLV for increased PT-INR and hyperbilirubinemia

	Cutoff value	Increased PT-INR		Hyperbilirubinemia	
		Sensitivity (%)	Specificity (%)	Sensitivity (%)	Specificity (%)
ICGK-F	0.096	81.8 (54.6–100)	74.4 (61.5–87.2)	58.3 (33.3–83.3)	71.1 (55.3–84.2)
r-FLV	555.585	72.7 (45.5–100)	79.5 (66.7–92.3)	50.0 (25.0–75.0)	73.7 (60.5–86.8)
ICGK × r-FLV	80.248	63.6 (36.4–90.9)	97.4 (92.3–100)	58.3 (33.3–83.3)	97.4 (92.1–97.4)

The figures in parentheses refer to 95% confidence interval

*PT-INR* international normalized ratio of prothrombin time, *ICGK* plasma clearance rate of indocyanine green, *ICGK-F* future liver remnant plasma clearance rate of indocyanine green, *r-FLV* functional liver volume of remnant liver

## Discussion

This study evaluated the predictive ability of the combined ^99m^Tc‑GSA SPECT/CT volume and ICGK for PHLF compared with ICGK-F obtained from ordinary CT volumetry. In the comparison of the AUC values, ICGK × r-FLV was the only index significantly greater than ICGK-F, with a significantly higher specificity for predicting increased PT-INR and hyperbilirubinemia. This result suggests that ICGK × r-FLV is a more specific and reliable predictor for PHLF than ICGK-F.

We used volume based on SUV threshold from ^99m^Tc‑GSA SPECT/CT images to measure remnant liver volume instead of volume measured from CT. The utility of the volume based on the SUV threshold has been reported for ^18^F-FDG-PET/CT and SPECT/CT [11–19]. Volume based on the SUV threshold reflects the degree of accumulation and heterogeneity of the target. In other words, the volume based on the SUV threshold is not just a morphological volume but also a functional volume, which is different from the volume measured using CT or MRI. Several studies have reported that MTV has higher predictability than tumor volume from MRI in tumor response to neoadjuvant chemotherapy in osteosarcoma [21, 22]. For ^99m^Tc-GSA SPECT/CT, one study investigated the utility of liver volume based on the SUV threshold [23]. However, this study investigated whole-liver function rather than remnant liver function. Several studies also have reported utility for combination of ICG clearance test and ^99m^Tc-GSA scintigraphy [24, 25]. However, these studies used the ratio of remnant liver volume to whole-liver volume and not the absolute remnant liver volume. The novelty of this study is the use of the absolute functional remnant liver volume based on the SUV threshold and the combination of the ICG clearance test as a quantitative whole-liver excretion function.

We considered the reasons for the high predictive ability of ICGK × r-FLV for PHLF. ICG is taken up into the liver by the same transporter as bilirubin [26, 27]. Therefore, ICG clearance test results were associated with bilirubin metabolism. In contrast, ^99m^Tc-GSA interacts with ASGPR, which is different from the bilirubin metabolic pathway. ASGPR levels decrease in patients with cirrhosis [6]. The number of hepatocytes also decreases in cirrhosis, while their function is maintained [28]. The degree of ^99m^Tc-GSA accumulation decreases in cirrhosis [7, 8]. Because r-FLV is the liver volume which reflects the liver accumulation of ^99m^Tc-GSA, r-FLV is associated with the number of hepatocytes, which is considered to reflect other hepatic functions different from bilirubin metabolism, such as protein synthesis. Therefore, a combination of the ICG clearance test and ^99m^Tc-GSA SPECT/CT can accurately evaluate the diversity of liver function.

In this study, the predictive ability of ICGK × r-FLV tended to be better than that of ICGK × r-FLV/t-FLV. If remnant liver volume is small, but its proportion of the total liver is high, r-FLV/t-FLV is calculated to be high. However, in this case, remnant liver function is not considered high, because remnant liver itself is small. Small remnant liver volume is an independent predictive factor for coagulation derangement [10]. On the other hand, r-FLV reflects remnant liver volume itself. Therefore, the ICGK × r-FLV data are considered superior to the ICGK × r-FLV/t-FLV data.

Using the same cut-off value for PHLF, the specificity of ICGK × r-FLV for increased PT-INR was significantly higher than that of ICGK-F, and r-FLV tended to be superior to ICGK in the evaluation of synthetic liver function. Similarly, using the same cut-off value for PHLF, the specificity of ICGK × r-FLV for hyperbilirubinemia was significantly higher than that of ICGK-F, and ICGK tended to be superior to r-FLV in the evaluation of liver function excretion. T-bilirubin reflects bilirubin metabolism and PT-INR reflects protein synthesis ability. Therefore, this result indicates that ICGK × r-FLV reflects both bilirubin metabolism and protein synthesis more accurately than ICGK-F. This result supported our hypothesis.

This study suggests that ICGK × r-FLV levels can predict PHLF with high sensitivity and specificity. We consider high specificity particularly important, because it implies low false positives. If ICGK × r-FLV was above the cut-off value, PHFL was considered unlikely. This indicates that ICGK × r-FLV can be used as a reliable indicator of safe hepatectomy.

This study has several limitations. First, this was a single-center study, and the number of patients was relatively small. Further multicenter studies with larger sample sizes are required to validate the results of this study. Second, most PHLF cases were classified as grade A, which was defined as PHLF that did not require clinical treatment. Therefore, this may lead to an overdiagnosis of PHLF. Third, not all the participants underwent hepatectomy. Therefore, the effects of selection bias cannot be excluded. Fourth, patient cohort was heterogeneous. There was no significant difference in the frequency of liver cirrhosis and etiology of liver disease between the PHLF and no PHLF group. However, the small number of patients in each category did not allow for subgroup analysis in each category. Further studies with larger sample sizes are required.

In conclusion, ICGK × r-FLV obtained from ^99m^Tc‑GSA SPECT/CT and ICG clearance test can be a reliable predictor for PHLF compared to ICGK-F obtained from ordinary CT volumetry.

## Data Availability

All imaging and assessment results were stored as electronic data. These data are available to the public upon request.
